# A Pan-Cancer Analysis of UBE2S in Tumorigenesis, Prognosis, Pathway, Immune Infiltration and Evasion, and Therapy Response from an Immune-Oncology Perspective

**DOI:** 10.1155/2022/3982539

**Published:** 2022-05-07

**Authors:** Haili Bao, Yi Luo, Guoshan Ding, Zhiren Fu

**Affiliations:** Department of Organ Transplantation, Shanghai Changzheng Hospital, Navy Medical University, Shanghai 200003, China

## Abstract

**Background:**

Ubiquitin conjugating enzyme E2S (UBE2S), a member of the ubiquitin-conjugating enzyme family, is known to play a pivotal role in tumorigenesis and progression in some tumor types. However, whether UBE2S plays an irreplaceable role in the immune-oncology context of tumorigenesis, prognosis, pathogenesis, immune regulation, and therapeutic response through certain common molecular mechanisms remains to be defined. The present pan-cancer study was intended to decipher the landscape of UBE2S in pathologic, immunological, and therapeutic aspects across various cancers.

**Methods:**

Data used for UBE2S analysis were obtained from TCGA database. The pan-cancer analysis was mainly focused on the expression patterns, prognostic values, mutation landscapes, biological pathways, tumor microenvironment remodeling, and therapeutic resistance of UBE2S using multiple databases including cBioPortal, Cancer Cell Line Encyclopedia (CCLE) database, Tumor Immune Estimation Resource (TIMER), and Gene Expression Profiling Interactive Analysis (GEPIA). External experimental validation was conducted to delineate the association of UBE2S with tumor phenotypes through assays of proliferation, colony formation, and migration. Data processing, statistical analysis, and plotting were performed using R software and GraphPad Prism software.

**Results:**

UBE2S was aberrantly expressed in almost all human cancers, and elevated UBE2S expression was unfavorably associated with the clinical pathological stage and prognosis. DNA methylation and RNA modification were significantly correlated with the UBE2S expression level. The results of enrichment analysis revealed that UBE2S positively regulated MYC, G2M cell cycle, and DNA repair pathways and negatively regulated adipogenesis, fatty acid metabolism, and heme metabolism. In addition, UBE2S exhibited a significantly positive correlation with myeloid-derived suppressor cell MDSC and Th2 subsets in almost all tumors analyzed. UBE2S could confer immune evasion via coexpressed immunoinhibitors and T cell exhaustion. Notably, a higher UBE2S expression indicated a higher level of stemness, TMB, MSI, and MMR deficiency and DNA methyltransferases, as well as chemotherapeutic resistance in various cancers. Notably, in vitro functional validation showed that UBE2S knockdown attenuated the phenotypes of proliferation, clonogenicity, and migration in hepatocellular carcinoma cells.

**Conclusions:**

Our study provided meaningful clues to support UBE2S as an immune-oncogenic molecule and shed light on potential applications of UBE2S in cancer detection, prognostic prediction, and therapeutic response assessment.

## 1. Introduction

The ubiquitin-proteasome system, mediated by E1 ubiquitin-activating enzymes, E2 ubiquitin-conjugating enzymes (E2s), and E3 ubiquitin ligases, governs key aspects of eukaryotic cell biological processes through a posttranscriptional protein modification mechanism [1]. Ubiquitin conjugating enzyme E2S (UBE2S), a well-established k11 linkage-specific E2 corresponding to a 24 kDa peptide [2], was reported to produce aberrantly higher expression and poor outcomes in multiple human cancers such as colorectal cancer (CRC), glioma, and hepatocellular carcinoma (HCC) [2–4]. Accumulative evidence has demonstrated that UBE2S plays a crucial role in mitosis and meiosis depending on the interaction with the anaphase-promoting complex/cyclosome (APC/C) for 26S proteasome-mediated protein degradation [5–7]. In addition, UBE2S was shown to regulate DNA damage-induced transcription silencing by modifying damaged chromatin through K11-linkage, and UBE2S downregulation suppressed nonhomologous end joining- (NHEJ-) mediated double-strand break (DSB) repair, contributing to improved chemotherapy sensitivity [8, 9]. Moreover, UBE2S overexpression promoted tumor cell proliferation, migration, and invasion through targeting degradation of p53 and the von Hippel-Lindau tumor suppressor [10, 11], and mutational inactivation of UBE2S attenuated malignant phenotypes [12]. Accordingly, UBE2S is generally identified as an oncogene, and targeting UBE2S may prove to be a potential therapeutic strategy. However, there is little knowledge about the pathogenic role of UBE2S in the immune-oncology context of the tumor microenvironment (TME), mutation burden, prognosis, and therapeutic response across different tumors.

In this study, we intended to conduct a visual analysis by using various bioinformatics algorithms at a systematic pan-cancer level to identify the expression patterns of UBE2S and determine the prognostic value of UBE2S across cancers. In addition, we tried to delineate the key signaling pathways with respect to cancer development and progression and decipher the correlation between UBE2S expression and immune infiltration, immune evasion, genome instability markers, and therapeutic response, hoping that the results could provide novel insights into the essential immune-oncology properties of UBE2S in the field of cancer detection, prognosis, and therapeutic response prediction.

## 2. Materials and Methods

### 2.1. Data Acquisition and Processing

The Cancer Genome Atlas (TCGA) database is a landmark cancer genomics project that molecularly characterizes over 20,000 primary cancer and matched normal samples spanning 33 cancer types [13]. In the present study, analytic data of the gene expression matrix, clinical information, tumor mutation burden (TMB), and microsatellite instability (MSI) were derived from TCGA database (http://cancergenome.nih.gov). In addition, the cBiopPortal database (http://www.cbioportal.org) was used to identify mutation landscapes of UBE2S in diverse cancers [14]. Transcripts per million (TPM) was employed throughout as an input format to quantify UBE2S expression.

### 2.2. Analysis of Gene Expression and Pathological Stage

Differential UBE2S expression between tumor tissues, para-cancerous tissues, and normal tissues was modeled using high-quality RNA sequencing datasets of 33 cancers from TCGA database (http://cancergenome.nih.gov), and a “box plot” together with a “paired dot plot” was employed for difference visualization. The differential expression of UBE2S across different pathological stages was visualized with violin plots based on the module of “stage plot” of the gene expression profiling interactive analysis, version 2 (GEPIA2, http://gepia2.cancer-pku.cn/#analysis).

### 2.3. Survival Prognosis of UBE2S

Prognostic parameters of overall survival (OS) and disease-specific survival (DSS) were analyzed to delineate the prognostic landscapes of UBE2S, with the R packages “survival” and “survminer” for Cox regression analysis. Kaplan-Meier curves and survival maps of UBE2S were drawn using the “Survival Analysis” module of GEPIA2 to further analyze and validate the prognostic value of UBE2S, with OS and DSS as the censored endpoints. A cutoff value of median UBE2S expression was selected to discriminate low- and high-expression cohorts.

### 2.4. Mutation Landscape, DNA Methylation, and RNA Modification of UBE2S

As mentioned above, the genomic mutation landscapes of UBE2S, including the mutation type, copy number alteration (CNA), and alteration frequency, were generated from the “cancer types summary” module of the cBioPortal database, and the corresponding mutated site of UBE2S protein was explored via the “mutations” module [14]. In addition, the fundamental regulation of DNA methylation and RNA modification on UBE2S expression was also investigated in the present study.

### 2.5. Gene-Related Enrichment Analysis

Based on the guilt-by-association principle, we explored the coexpression of UBE2S with other associated genes extracted from TCGA transcriptome matrix using Pearson's correlation. Sorted by the level of the association index between UBE2S and related genes, those genes at the top of the gene lists were chosen for enrichment analysis to predict the impact of UBE2S expression on biological processes. R package “GSVA” was used for Gene Set Variation Analysis (GSVA), and “clusterprofiler” was employed for Gene Set Enrichment Analysis (GSEA).

### 2.6. Analysis of Immune Infiltration and Immune Checkpoint Genes

The immune score and stromal score were introduced to determine the abundance of immune and stromal cells via R package of “ESTIMATE,” with a higher score referring a higher proportion of the respective component in TME [15]. ESTIMATE Score represented integrated both scores of immune and stromal cells and negatively correlated with tumor purity. Coexpression analysis of immune cell subtypes and UBE2S was performed with “CIBERSORT” package for infiltration score quantification, and four algorithms (EPIC, MCPCOUNTER, QUANTISEQ, and TIMER) were applied to calculate immune infiltration [16]. We further delineated the potential regulation of UBE2S on myeloid-derived suppressor cells (MDSCs), Th1 subsets, and Th2 subsets using “Immune-Gene” module of TIMER2.0 database.

To further determine the underlying mechanisms of immune evasion, correlations of UBE2S with immunomodulatory and immune checkpoint genes were calculated and presented as heatmaps via packages of “limma,” “reshape2,” and “RColorBrewer.” Analyzed immune checkpoint-related genes included CD276, VEGFB, TGFB1, VEGFA, IDO1, LAG3, PDCD1 (PD1), CD274, IL12A, TIGHT, IL10, HMGB1, CD80, ICOS, IL1, and TNFRSF. T cell exhaustion-related markers, including PDCD1, CTLA4, LAG3, TIGIT, CXCL13, and HAVCR2 genes, were deciphered. Gene expression data were normalized with log2 transformation.

### 2.7. Analysis of Genomic Alteration as well as Therapy Response

Subsequently, genome instability markers of homologous recombination deficiency (HRD), TMB, MSI, and mutational mismatch repair (MMR, including five definitive members of MLH1, MSH2, MSH6, PMS2, and EPCAM) were evaluated. In addition, DNA methylation methyltransferases (DNMT), including DNMT1, DNMT2, DNMT3A, and DNMT3B, were considered to govern a fundamental role in tumorigenesis and disease progression. Therefore, we explored the landscapes of DNA methyltransferases. All datasets were obtained from TCGA database, and the results were presented based on Pearson's method.

The half maximal inhibitory concentration (IC50) was introduced to describe the correlation between CCNA2 expression and specific therapy response in cancer cell lines. Elevation of IC50 denoted that high expression of UBE2S may confer multidrug resistance. The datasets were obtained from Cancer Cell Line Encyclopedia database (CCLE, https://sites.broadinstitute.org/ccle).

### 2.8. Cell Culture and Transfection

HepG2 cell line, Hep3B cell line, Bel-7404 cell line, and SMMC-7721 cell line were obtained from the National Key Laboratory of Medical Immunology & Institute of Immunology, Navy Medical University. MHCC-97H cell line and QGY-7703 cell line were obtained from Eastern Hepatobiliary Surgery Hospital of Navy Medical University. All cell lines were cultured in DMEM medium supplemented with 10% fetal bovine serum, 1% penicillin, and streptomycin in 5% CO_2_ at 37°C. Cells were transfected with siRNA using Lipofectamine 2000 (Invitrogen) according to the manufacturer's instructions. Cells were transiently transfected for 48 h for mRNA and protein assessments. Sequences for siRNAs were as follows: siCtrl sense UUCUCCGAACGUGUCACGUTT, antisense ACGUGACACGUUCGGAGAATT; siUBE2S-1 sense UCAUCCGCCUGGUGUACAATT, antisense UUGUACACCAGGCGGAUGATT; and siUBE2S-2 sense GACACGUACUGCUGACCAUTT, antisense AUGGUCAGCAGUACGUGUCTT.

### 2.9. Quantitative Real-Time PCR

cDNA was synthesized from the total RNA, using the PrimeScript RT reagent Kit (TAKARA, RR036A) according to the manufacturer's instructions. qRT-PCR was performed with SYBR Premix ExTaq (TAKARA, RR420A). The expression of *β*-actin expression was used as a reference control. The primers were as follows: UBE2S, forward: 5′-ACAAGGAGGTGACGACACTGA-3′ and reverse: 5′-CCACGTTCGGGTGGAAGAT-3′; *β*-actin, forward: 5′-TGGCACCCAG-CACAATGAA-30 and reverse: 5′-CTAAGTCATAGTCCGCCTAGAAGCA-3′.

### 2.10. Western Blotting

Proteins extracted from HCC cells were quantified with a BCA protein assay kit (Thermo Fisher Scientific, USA). Then, protein samples were fractionated by SDS-PAGE, transferred to PVDF membranes, and incubated with a primary specific antibody for UBE2S (1 : 1000, #ab177508, Abcam) and *β*-actin (1 : 5000, #SD0034, Shanghai SIMUWU technology). After being washed by TBST, the membranes were incubated with corresponding secondary antibody. ECL detection reagent (Vazyme, China) was used to visualize the protein bands.

### 2.11. Cell Proliferation Assays

MHCC-97H cells with or without UBE2S silencing were seeded into 96-well plates (100 *μ*l cell suspensions). Cell numbers were assessed every 24 h by CCK-8 assays according to the manufacturer's instructions.

### 2.12. Colony Formation

After treatment with siRNA, MHCC-97H cells were collected and seeded in 6-well plate at a density of 1.0 × 10^3^ per well and then incubated at 37°C for 5 days. Colonies were fixed with methanol, stained with 0.1% crystal violet, and counted.

### 2.13. Cell Migration Assays

Cell migration assays were performed in 24-well plates with polyethylene terephthalate membrane filters separating the lower and upper culture chambers. MHCC-97H cells (2 × 10^4^) were added to the upper chambers in serum-free DMEM medium, and 10% FBS was added to the lower chambers. After 24 h, chambers were fixed with methanol for 15 min and stained with 0.1% crystal violet for 20 min and imaged.

### 2.14. Statistical Analysis

R software (version 4.1.3) was used for data processing, statistical analysis and plotting. Differences between two groups and multiple groups were analyzed using default Wilcoxon's test and one-way analysis of variance (ANOVA), respectively. Differences in OS and disease-free survival (DFS) between the high- and low-expression groups were calculated by Kaplan-Meier analysis and log-rank test. Hazard ratios (HRs) were determined by univariate and multiple Cox regression analysis. Correlation analysis of clinicopathological stages, immune checkpoint expression, immune infiltration abundance, and genomic mutation with UBE2S was performed using Pearson's correlation test. All experiments were independently repeated at least three times. Data are expressed as the mean ± SEM. All data were analyzed using Graph Prism (ver 8.0, GraphPad Software, La Jolla, CA, USA) and assessed using a *t*-test between the two groups and one-way ANOVA tests for three or more groups. The results were deemed significantly different with two-sided *p* < 0.05.

## 3. Results and Discussion

### 3.1. Results

#### 3.1.1. Elevation of UBE2S Expression Is Featured across Human Cancers

**Figure 1:**
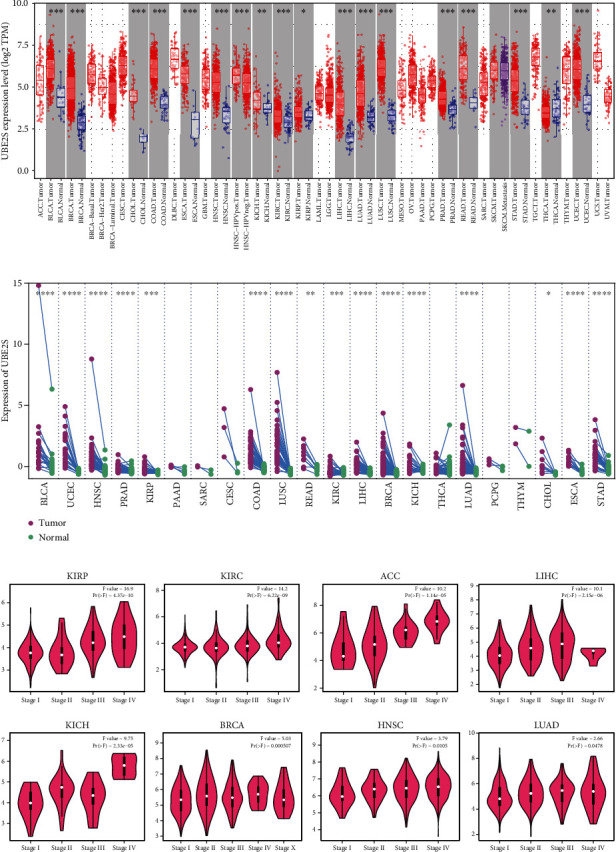
UBE2S expression pattern in cancer and tumor tissues. (a) Divergence in UBE2S expression across cancer and normal tissues of TCGA was determined by TIMER2. (b) Divergence in UBE2S expression across cancer and corresponding para-cancerous tissues from TCGA database. ^∗^*p* < 0.05;  ^∗∗^*p* < 0.01;  ^∗∗∗^*p* < 0.001. (c) Based on GEPIA online tool, pathological stage characterizations were identified by UBE2S expression in KIRP, KIRC, ACC, LIHC, KICH, BRCA, HNSC, and LUAD of TCGA. TCGA: The Cancer Genome Atlas; KIRP: kidney renal papillary cell carcinoma; KIRC: kidney renal clear cell carcinoma; ACC: adrenocortical carcinoma; LIHC: liver hepatocellular carcinoma; KICH: kidney chromophobe; BRCA: breast invasive carcinoma, HNSC: head and neck squamous cell carcinoma; LUAD: lung adenocarcinoma.

To unambiguously identify UBE2S differential expression in human cancers, high-quality RNA sequencing data from the Cancer Genome Atlas (TCGA) were employed in this study. In the nonpaired sample cohort, the expression abundance of UBE2S in tumor samples was significantly higher than that in the corresponding normal samples (Figure 1(a)), including bladder urothelial carcinoma (BLCA), cholangiocarcinoma (CHOL), colon adenocarcinoma (COAD), esophageal carcinoma (ESCA), head and neck squamous cell carcinoma (HNSC), kidney renal clear cell carcinoma (KIRC), liver hepatocellular carcinoma (LIHC), lung adenocarcinoma (LUAD), lung squamous cell carcinoma (LUSC), prostate adenocarcinoma (PRAD), rectum adenocarcinoma (READ), stomach adenocarcinoma (STAD), uterine corpus endometrial carcinoma (UCEC) (all *p* < 0.001), kidney renal papillary cell carcinoma (KIRP) (*p* < 0.05), and kidney chromophobe (KICH) as well as thyroid carcinoma (THCA) (*p* < 0.01). Of note, although no controlled tissues were available in the remaining tumor types including adrenocortical carcinoma (ACC), breast invasive carcinoma (BRCA), and glioblastoma multiforme (GBM), elevated expression abundance of UBE2S could still be observed. In the paired sample cohort, a similar pattern of increased UBE2S expression was visible in most tumor tissues compared with the para-cancerous tissues, including BLCA, UCEC, HNSC, KIRP, COAD, LUSC, READ, KIRC, LIHC, BRCA, KICH, LUAD, CHOL, ESCA, and STAD (Figure 1(b)). Beyond the above delineations, we analyzed the correlation between UBE2S expression profiles and pathological stages across 33 cancers of TCGA. The results demonstrated that UBE2S overexpression was significantly correlated with an advanced pathological stage in KIRP, KIRC, ACC, LIHC, KICH, BRCA, HNSC, and LUAD (all *p* < 0.05) (Figure 1(c)). Given the high UBE2S expression in high-pathological stages, we speculated that UBE2S might be involved in facilitating cancer progression and metastasis in these cancers.

#### 3.1.2. UBE2S Overexpression Indicates Unsatisfied Prognosis

**Figure 2:**
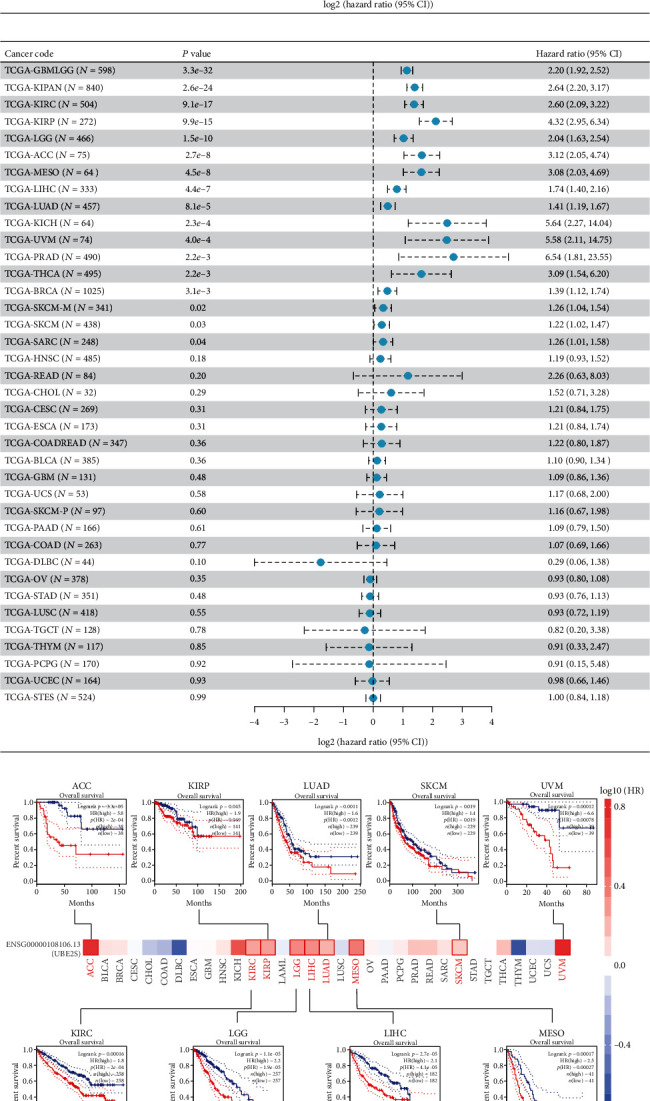
Multifaceted prognostic analysis of UBE2S at pan-cancer level. (a) The association of UBE2S with OS and DSS in pan-cancers from TCGA database. (b) Survival maps and survival curves of OS (c) and DFS (d) were delineated to elaborate and validate UBE2S prognostic values using GEPIA2 tool.

Subsequently, we focused on the pan-cancer analysis of the impact of UBE2S expression profiles on survival using Cox regression analysis. As shown in Figure 2(a), a high level of UBE2S expression was associated with a worse prognosis in terms of OS in LIHC, ACC, KIRP, LUAD, KICH, PRAD, THCA, KIRC, GBMLGG (Glioma), KIPAN (Pan-kidney cohort, KICH+KIRC+KIRP), brain lower grade glioma (LGG), mesothelioma (MESO), uveal melanoma (UVM), skin cutaneous melanoma (SKCM), and sarcoma (SARC). Meanwhile, to remove the bias of nontumor-related death factors, DSS was employed as the censored endpoint, and multiple cancers with higher UBE2S expression exhibited worse outcomes (Figure 2(b)). In addition, plots for survival of several cancers were delineated to capture and validate the influential pattern of UBE2S by Kaplan-Meier method. The results demonstrated that elevated UBE2S expression significantly contributed to worse OS in ACC, KIRP, LUAD, SKCM, UVM, KIRC, LGG, LIHC, and MESO (all *p* < 0.05) (Figure 2(c)). Besides, a similar prognostic pattern was also obtained when DFS was employed as the censored endpoint, including ACC, KIRC, LGG, PRAD, BLCA, KIRP, and LIHC (all *p* < 0.05) (Figure 2(d)). Taken together, UBE2S expression was negatively correlated with survival across different human cancers.

### 3.2. Mutation Landscape of UBE2S in Cancers

**Figure 3:**
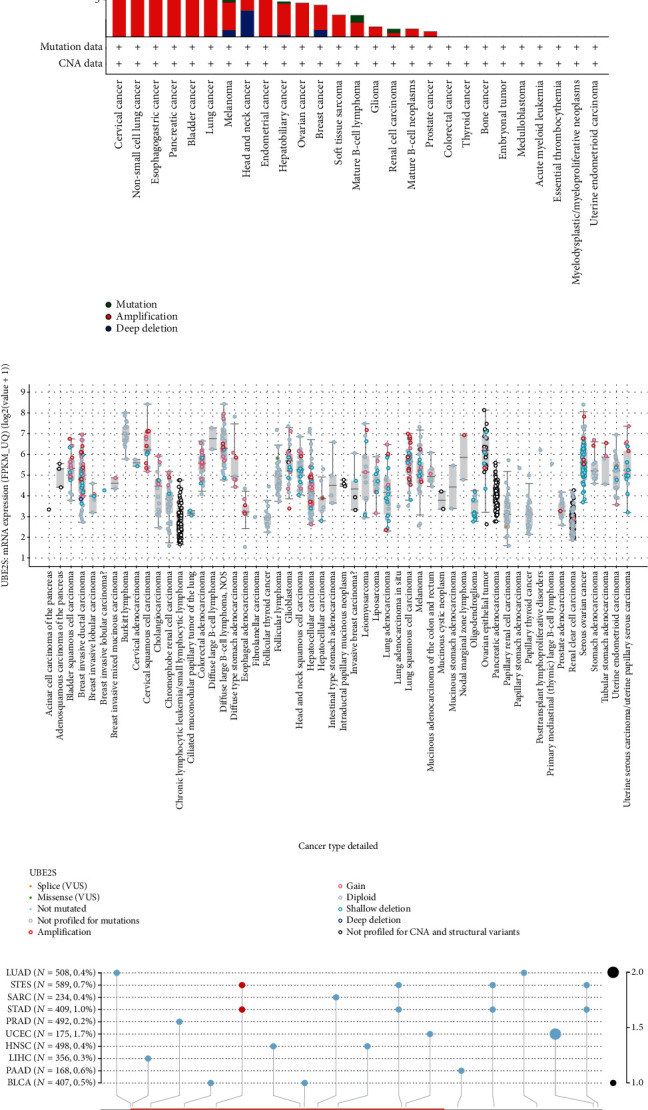
Mutation landscape of UBE2S in cancers. UBE2S alteration frequency with specific types (a), general mutation counts (b), and protein mutation sites (c) were depicted across different tumor types.

The genetic-alteration profiles of UBE2S across multiple tumors of TCGA datasets were deciphered (Figures 3(a) and 3(b)). UBE2S harbored very high genetic-alteration frequencies (>10%) in cervical cancer, non-small-cell lung cancer, esophagogastric cancer, and bladder cancer. Notably, most of the analyzed cancers held the only genetic alteration of amplification, and copy number deletion accounted for the highest occupation (>50%) in head and neck cancer (Figure 3(a)). In addition, genetic alteration types of amplification, gain and shallow deletion were widely observed at the mRNA level of UBE2S (Figure 3(b)). In addition, a total of 16 variant sites, involving 15 missense mutations and 1 splice mutation were identified, and 11 mutations were detected in the UBCC domain (Figure 3(c)). UCEC presented a relatively high mutation frequency (1.7%).

### 3.3. Correlation of UBE2S Expression with DNA Methylation and RNA Modification

**Figure 4:**
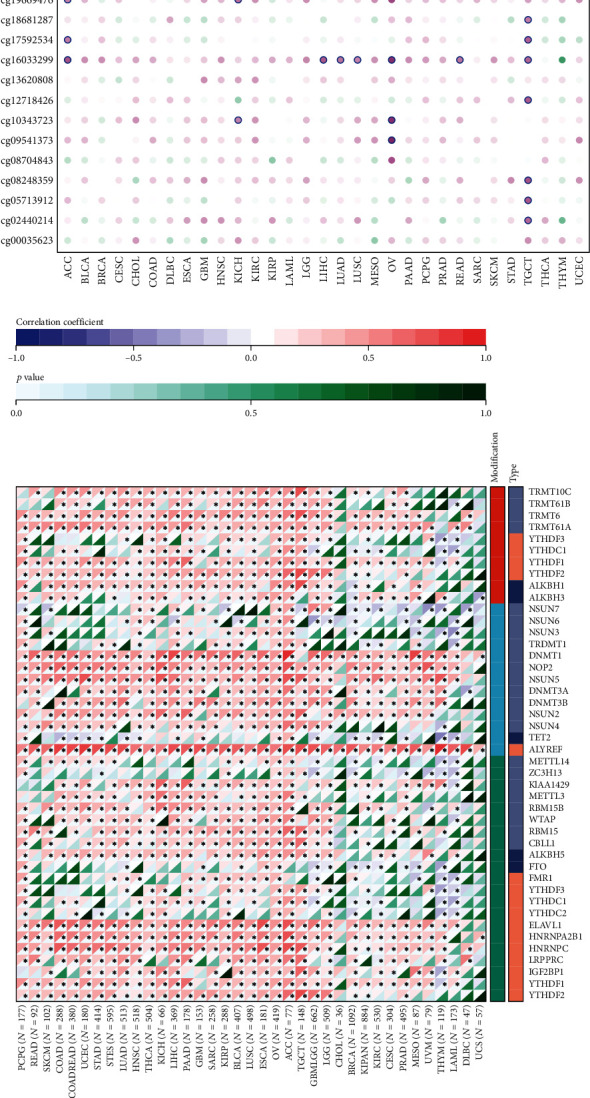
Correlation of UBE2S expression with DNA methylation and RNA modification in pan-cancers. (a) UBE2S expression patterns under various DNA-methylation loci through different probes. (b) The association between UBE2S and RNA modification related genes (including m1A, m5C, and m6A).

To explore whether UBE2S expression was subject to regulation by DNA methylation and RNA modification, we first determined UBE2S expression patterns under various DNA-methylation loci through different probes. Our results demonstrated that DNA methylation could exert a negative modulation on UBE2S expression in multiple cancers (Figure 4(a)). For instance, significantly decreased UBE2S expression was regulated by DNA methylation patterns of different loci in TGCT via probes cg26692860, cg24973886, cg23437684, and cg23373897. In addition, consistent trends between UBE2S and RNA modification related genes (including m1A, m5C, and m6A) were also observed in most analyzed tumors (Figure 4(b)), suggesting that UBE2S expression might be modulated at a pervasive level of RNA posttranscriptional modification.

### 3.4. Identification of the Key Signaling Pathways of UBE2S

**Figure 5:**
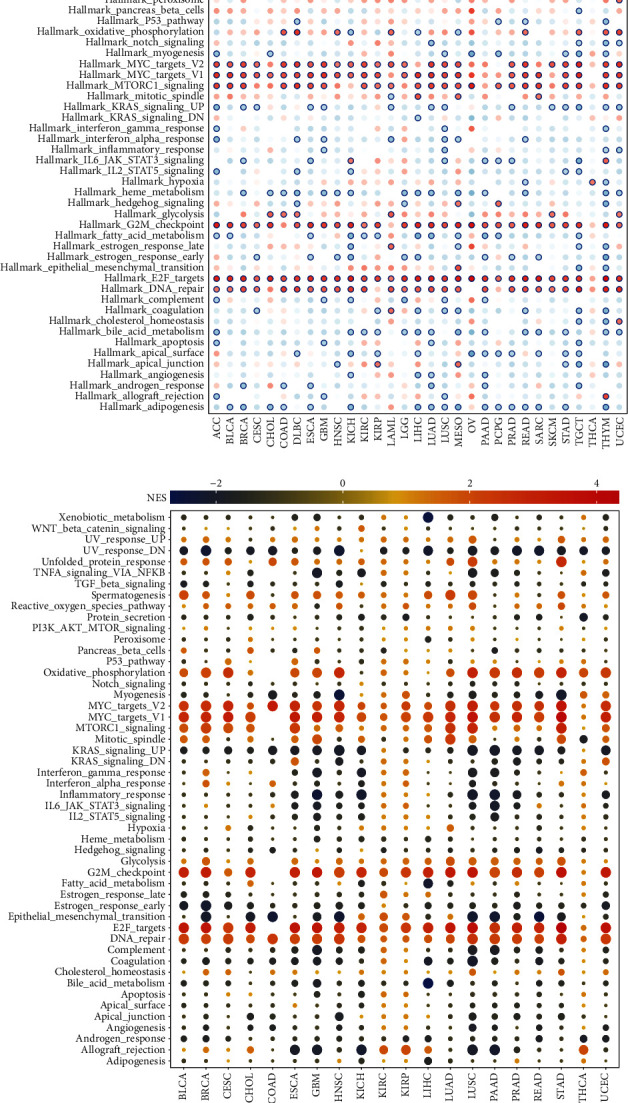
Enrichment analyses and visualization of UBE2S-related partners based on TCGA dataset. UBE2S-related signaling pathways were determined by GSVA (a) and GSEA (b) algorithms, respectively, in pan-cancers. GSVA: Gene Set Variation Analysis; GSEA: Gene Set Enrichment Analysis.

To gain more insights into the biological function of UBE2S in cancers, we carried out GSVA and GSEA analysis to identify underlying mechanisms and relevant pathways by calculating the enrichment scores of canonical tumor-associated pathways at the pan-caner level (Figures 5(a) and 5(b)). The results showed that UBES2 could stimulate oncogenic pathways, including MYC, MTORC1 signaling, G2/M checkpoint, E2F signaling, and DNA repair pathways, which indicated that UBE2S played a substantial role in the tumorigenesis and progression of human cancers (Figure 5(a)). In contrast, UBE2S negatively regulated adipogenesis, bile acid metabolism, fatty acid metabolism, and heme metabolism (Figure 5(a)) but positively regulated oxidative phosphorylation (Figure 5(b)), suggesting that UBE2S may participate in tumor-associated metabolic pathways. Our results also showed that UBE2S was involved in nearly all the listed canonical hallmarks in TGCT and THYM, implying the importance of UBE2S in tumorigenesis (Figure 5(a)). Collectively, these data suggest that UBE2S may be implicated in the occurrence and progression of tumors through affecting cell cycle and metabolism and show great potential for targeting UBE2S for tumor therapeutic intervention.

### 3.5. Negative Regulation of UBE2S on the Immune Microenvironment

**Figure 6:**
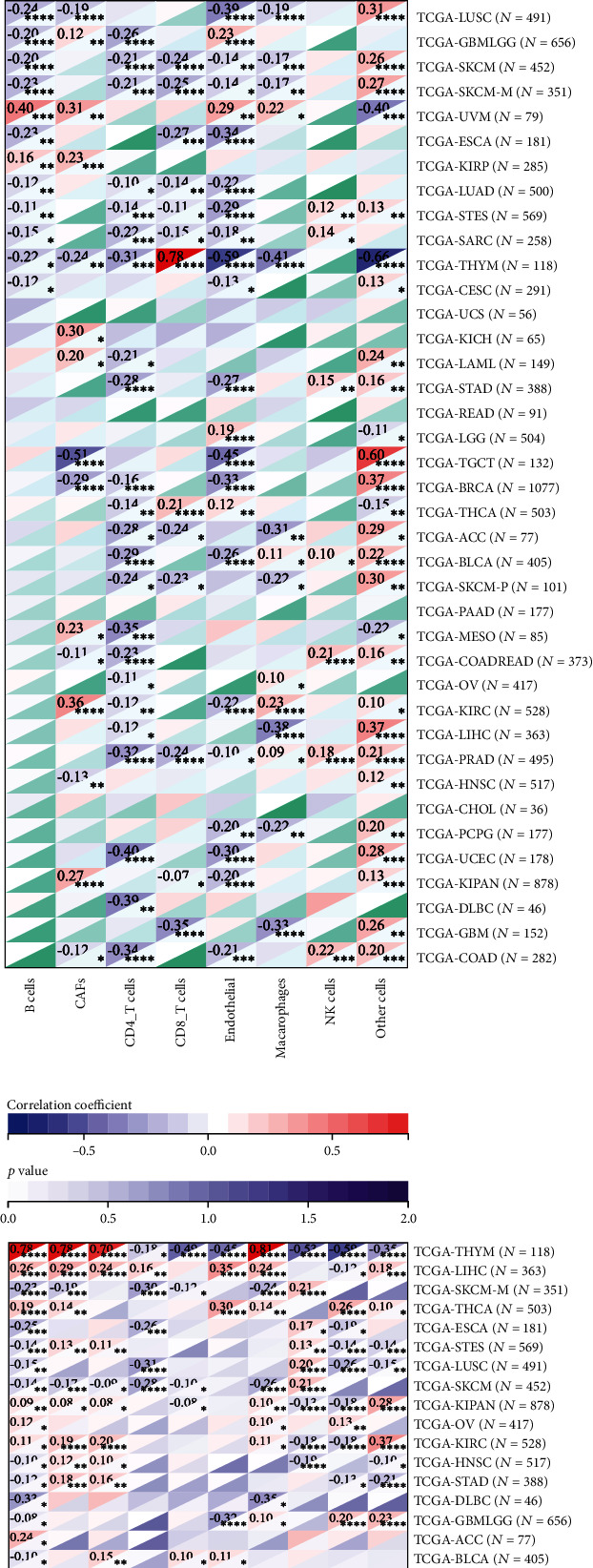
Correlation of UBE2S with immune and stromal cell infiltration across TCGA cancers. Respective scatter plot of UBE2S with immune score (a) and stromal score (b) and ESTIMATE Score (c) were adopted to characterize the overall variation tendency of immune cells, stromal cells, and tumor purity at the pan-cancer level. Four algorithms of EPIC (d), MCPCOUNTER (e), QUANTISEQ (f), and TIMER (g) were applied to evaluated specific immune and stromal subsets. The correlation of UBE2S expression with MDSC, Th1, and Th2 subsets of CD4+ T cells (h).

The immune score and stromal score were used to identify the association between tumor immune infiltration and UBE2S expression profiles across 33 cancer types. As shown in Figure 6(a), UBE2S expression was negatively correlated with the immune score in ACC, LUAD, and STAD and positively correlated with the immune score in KIRC, LGG, and THYM, indicating that a higher UBE2S expression level was associated with a lower immune cell infiltration rate in TME. Additionally, the UBE2S expression level was negatively correlated with stromal score in most cancers including ACC, LIHC, LUAD, and COAD and positively correlated with stromal score in KIRC (Figure 6(b)). Besides, a similar negative relationship between UBE2S expression and ESTIMATE Score was also observed in most cancer types (Figure 6(c)).

To further investigate the UBE2S landscape in TME, quantitative analysis of infiltrating immunoreactive cells of diverse cancers was carried out using algorithms of EPIC, MCPCOUNTER, QUANTISEQ, TIMER, XCELL, and TIDE. The results showed a significant negative relationship between macrophage infiltration and UBE2S expression in THYM, GBM and LUSC (Figures 6(d)–6(g)). The subgroup analysis of macrophages based on QUANTISEQ algorithms showed that elevated UBE2S expression positively regulated the infiltration of M1 subgroup in BLCA, COAD, READ, and HNSC and negatively regulated the infiltration of M2 subgroup in THYM, BRCA, and LUAD (Figure 6(e)). Furthermore, a significant negative regulatory effect on B cell recruitment was also observed in THYM, SKCM, metastatic SKCM, and LUAD. Interestingly, UBE2S seemed to exert a positive regulatory effect on CD8+ T cell infiltration, especially in THYM (Figures 6(d)–6(g)). Notably, increased UBE2S abundance reduced the level of immune cell recruitment in THYM and enhanced the level of immune cell infiltration in LIHC (Figures 6(d)–6(g)).

Furthermore, we deciphered infiltration changes of myeloid-derived suppressor cells (MDSC) and CD4+ T cells via TIDE and XCELL algorithms. The heatmap depicted that UBE2S exhibited a statistically positive correlation with MDSC and Th2 subsets in almost all analyzed tumors (Figure 6(h)). Intriguingly, UBE2S expression was also positively relevant to the infiltration abundance of Th1 subsets. Overall, UBE2S was definitely responsible for immune and stromal cell infiltration in most human cancers and played a prominent role in immunooncological interaction.

### 3.6. UBE2S Is Associated with Tumor Immune Evasion via Coexpression of Immunoinhibitors and T Cell Exhaustion

**Figure 7:**
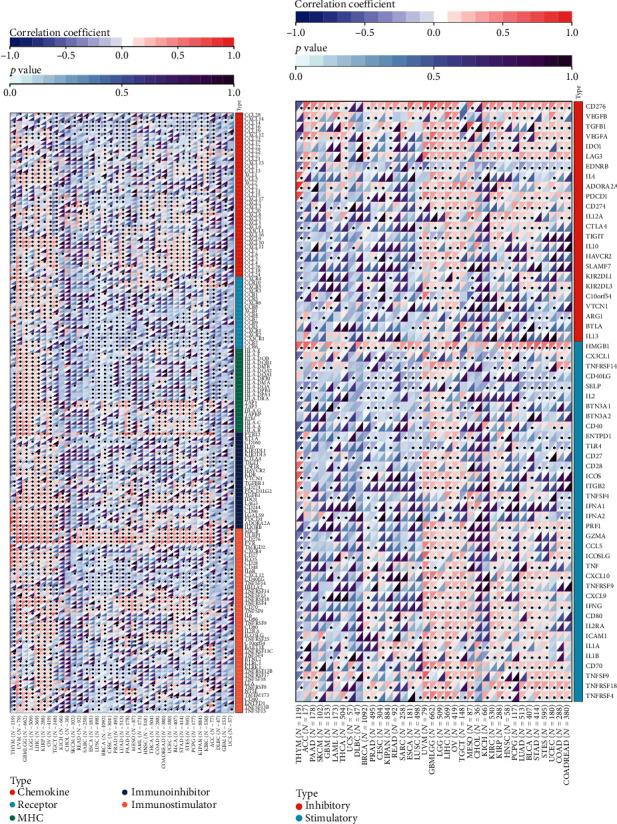
Correlation of UBE2S expression level with immunomodulatory genes and immune checkpoint genes. The heatmap of correlation between UBE2S expression and immunomodulatory genes (covering chemokine, immune receptor, MHC molecule, immunoinhibitor, and immunostimulator) (a) and immune checkpoint genes (b) based on TCGA dataset.

As described above, UBE2S plays a significant role in immune infiltration across diverse cancers. To further discover the in-depth landscape of immune regulation, we investigated the relationship between UBE2S expression and immune genes including immunomodulatory and immune checkpoint genes (Figures 7(a) and 7(b)). USBE2S exerted a positive regulatory effect on chemokines, immune receptors, MHC, immunoinhibitors, and immunostimulators in UVM, GBMLGG, LGG, LIHC, KIRP, and OV, which was in contrast to the negative regulatory effect in ESCA, LUSC, and BRCA (Figure 7(a)). Besides, MICB, ULBP1, CD276, and PVR were found to be upregulated significantly in almost all analyzed tumors, indicating that UBE2S could enable multiple human tumors to progress and escape immune surveillance via different immune-oncology mechanisms (Figure 7(a)). Our pooled analysis for immune checkpoint genes showed that UBE2S could positively exert a regulatory effect on several immunoinhibitors of immune evasion, including CD276, IDO1, LAG3, PDCD1, CD274, CTLA4, and TIGIT in LIHC, OV, TGCT, KIRP, and BLCA, and displayed a negative relationship with most immunoinhibitors in THYM (Figure 7(b)). Remarkably, the abundance of T cell-depleted markers (PDCD1, CTLA4, LAG3, and TIGIT) increased with UBE2S expression. In summary, UBE2S might play a crucial role in immune evasion through modulating immunomodulatory genes as well as T cell-exhaustion marker genes, suggesting that targeting UBE2S may improve the efficacy of cancer immunotherapy.

### 3.7. UBE2S Overexpression Indicates a Higher Level of Stemness, TMB, MSI, HRD, and MMR Deficiency and DNA Methyltransferases, as well as Therapeutic Resistance

**Figure 8:**
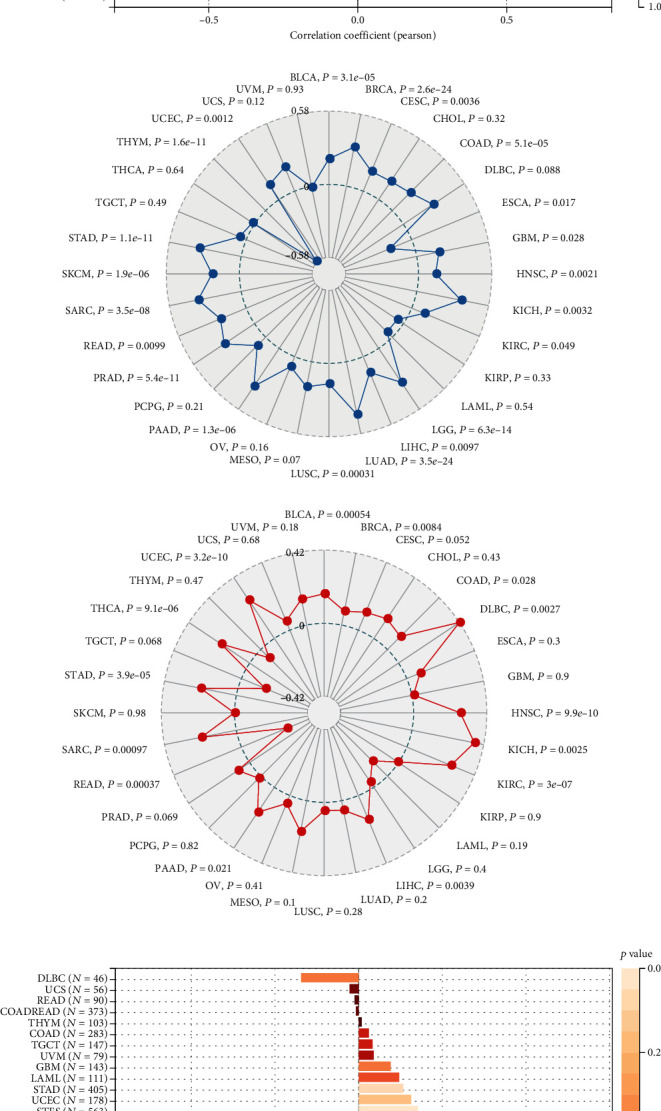
Analyses of tumor stemness, TMB, MSI, MMR deficiency, DNA methyltransferases, and therapeutic resistance. The correlation of UBE2S expression with tumor stemness was evaluated on three dimensions: DMPss (a), DNAss (b), and RNAss (c). Radar maps of correlation of UBE2S with TMB (d) and MSI (e). (f) Bar graph of correlation between UBE2S and HRD. Variation tendencies of five MMR genes (g) and four methyltransferases (h). (i) IC50 values of multiple drugs between UBE2S high- and low-expression groups obtained from CCLE database.

Stemness acquisition or cell transformation and maintenance of stem-cell-like characteristics has been proven to enhance the capacity of heterogeneity, tumor initiation, proliferation, metastasis, drug resistance, and TME formation via various intrinsic and extrinsic factors [17]. Therefore, we attempted to delineate the correlation between UBE2S expression and stemness based on three stemness indices: differentially methylated probe-based stemness scores (DMPss), DNA methylation-based stemness scores (DNAss), and RNA expression-based stemness scores (RNAss). Our results showed that UBE2S expression was significantly and positively correlated with DMPss and DNAss in LGG, TGCT, and GBMLGG (*r* > 0.4, *p* < 0.05) and negatively correlated with them in THYM (*r* < −0.5, *p* < 0.05) (Figures 8(a) and 8(b)). Furthermore, a positive correlation was observed between UBE2S expression and RNAss in READ, LUAD, UCEC, DLBC, BRCA, TGCT, and THYM (*r* > 0.5, *p* < 0.05) (Figure 8(c)).

Growing evidence implicates that TMB is a promising biomarker for tumor-specific response to immunotherapy, especially immune checkpoint blockade [18–20]. In our analyzed radar chart, the degree of TMB was markedly positively associated with UBE2S expression in BLCA, BRCA, KICH, LIHC, LUAD, COAD, READ, and STAD and negatively associated with it in THYM (Figure 8(d)).

In addition to TMB analysis noted above, we also analyzed genome instability markers involved in HRD, MSI, and mutational MMR [21] and found that UBE2S was positively correlated with MSI in BLCA, BRCA, COAD, HNSC, KICH, KIRC, LIHC, and UCEC and negatively correlated with MSI in THYM and READ (Figure 8(e)). A consistent positive trend was observed between UBE2S and HRD in LIHC, BLCA, LUAD, KIRC, SARC, KICH, ACC, BRCA, and KIRP. Notably, no significant negative correlation was observed across various cancers (Figure 8(f)).

The variation tendencies of five MMR genes are illustrated in Figure 8(g), showing that UBE2S was positively correlated with MLH1 in 15 cancer types, MSH2 in 18 cancer types, MSH6 in 21 cancer types, PMS2 in 4 cancer types, and EPCAM in 10 cancer types. In contrast, a negative correlation of CCNA2 with EPCAM and PMS2 was observed in THYM and READ. DNA methylation is thought to be implicated in the regulation of gene expression, diverse bioprocesses such as epigenetic modification, or even tumor response to immunotherapy [22, 23]. We analyzed four methyltransferases (DNMT1~4). The results in Figure 8(h) showed that there was a significantly positive correlation between UBE2S expression and all four methyltransferases in 5 cancer types (KICH, LGG, LIHC, STAD, and BLCA). In contrast, only 3 cancer types (PCPG, CESC, and CHOL) were not associated with any of the four methyltransferases. Taken together, UBE2S expression may play a vital role in TME formation across various cancers.

Subsequently, we analyzed the correlation between UBE2S expression and chemical drug sensitivity based on the CCLE database. The results showed that elevated UBE2S expression could give rise to a higher IC50 of chemical drugs (Figure 8(i)). In other words, elevated UBE2S expression could confer multidrug resistance to human tumors, such as lapatinib (targeting EGFR), TAE684 (targeting ALK), L-685458 (targeting GS), and erlotinib (targeting EGFR) (Figure 8(i)).

### 3.8. UBE2S Promotes Cell Proliferation and Migration in HCC

**Figure 9:**
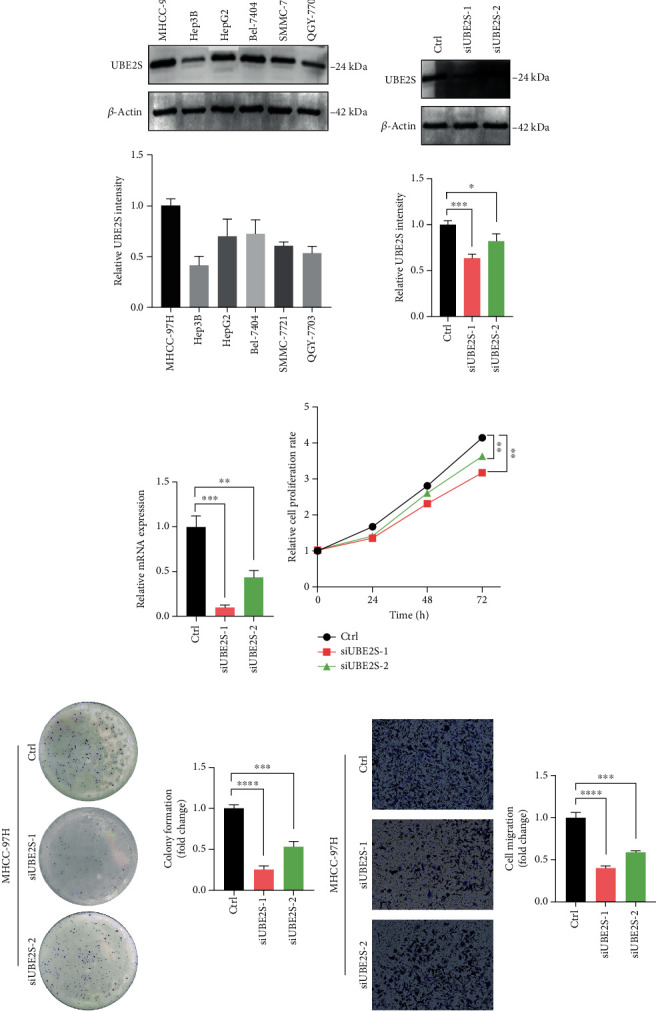
UBE2S promotes cell proliferation and migration in HCC. (a) UBE2S protein levels in different HCC cell lines were determined by western blot. Western blotting (b) and qRT-PCR (c) analysis of UBE2S in MCHH-97H cells transfected with siRNA. (d) Effects of UBE2S on cell proliferation were determined by CCK-8. (e) Cells with or without UBE2S were cultured for 5 days. Colonies were counted and indicated. (f) Transwell assays were performed to evaluate the effect of UBE2S on the cell migration. ^∗^*p* < 0.05,  ^∗∗^*p* < 0.01,  ^∗∗∗^*p* < 0.001, and^∗∗∗∗^*p* < 0.0001.

To further delineate the landscape of UBE2S in tumor proliferation and progression, we carried out experimental validation. First, the protein expression levels of UBE2S were determined in six human HCC cell lines. Results confirmed that UBE2S protein was ubiquitously expressed in HCC cells, and MHCC-97H cells exhibited the highest expression (Figure 9(a)). Consequently, MHCC-97H cell was selected for the subsequent experiments. UBE2S was knocked down by siRNA in MHCC-97H cell, and satisfactory knockdown efficiency of UBE2S was detected by western blotting and qRT-PCR (Figures 9(b) and 9(c)). CCK-8 results showed that UBE2S silencing inhibited HCC cell proliferation (Figure 9(d)). Besides, colony formation assays presented that UBE2S knockdown attenuated the ability of clonogenicity of MHCC-97H cells (Figure 9(e)). Similarly, a repressed migration phenotype of MHCC-97H cell was observed through transwell assay (Figure 9(f)).

## 4. Discussion

UBE2S can catalyze ubiquitin transfer to substrates for protein degradation via either E3-dependent or E3-independent mechanisms [24]. Previous works have illustrated that distinct UBE2S elevation is harbored in multiple human cancers, and aberrant UBE2S expression is responsible for carcinogenicity, cell cycle disruption, drug resistance, and attenuation of ischemia-reperfusion injury (IRI) [9, 25–27]. Although studies have provided some insights into the oncogenic biological function of UBE2S, there is no comprehensive delineation regarding whether and how UBE2S determines oncogenesis, progression, and metastasis in various cancers, and therefore, a systematical pan-cancer analysis is warranted to elucidate the role of UBE2S in the immune-oncology context of TME, mutation burden, prognosis, and therapeutic response. Using the high-quality RNA sequencing data from TCGA, we demonstrated that UBE2S played a prominent role in the immune-oncology setting and may prove to be a predictive biomarker for prognosis, immune infiltration, and therapy response.

In this pan-cancer study, we first delineated the mRNA expression profile of UBE2S and its correlation with the tumor pathological stage. Compared with normal or para-carcinoma tissues, UBE2S expression was found to be distinctly higher in a series of cancers, suggesting that UBE2S may play an oncogenic role across these cancers. Furthermore, the aggressive characteristics of UBE2S were supported by the correlation between the upregulated expression of UBE2S and an advanced clinical stage in KIRP, KIRC, ACC, LIHC, KICH, BRCA, HNSC, and LUAD. With respect to prognostic paradigms, the high expression of UBE2S was linked with worse OS, PFS, DSS, and DFS in multiple cancers. The survival landscape was also validated by employing the GEPIA2 database, and results denoted that UBE2S overexpression was associated with unsatisfactory OS and DFS in LIHC, ACC, KIRC, and LGG. The above results regarding the oncogenic and prognostic paradigms of UBE2S are consistent with the previous experimental studies, confirming that UBE2S plays a vital carcinogenic role independent of the algorithms and databases [2, 27, 28].

Previous works have shown that elevated UBE2S expression promoted the proliferation, invasion, metastasis, and G1/S phase transition of cell cycle in HCC through interacting with TRIM28 [4], and decreased UBE2S expression recovered drug sensitivity in GBM via suppressing NHEJ-mediated DSB repair [9]. In addition to reported pathways involving DNA repair, G2/M checkpoint, PTEN/AKT, and MYC, our enrichment analysis also discovered the potential biological role of UBE2S in E2F signaling and MTORC1 signaling through GSVA and GSEA algorithms. It is interesting to note that UBE2S also played a substantial role in regulating adipogenesis, bile acid metabolism, fatty acid metabolism, and heme metabolism, and oxidative phosphorylation in our results, implying that UBE2S might induce malignant phenotypes via tumor-associated metabolic pathways. Additional experimental studies are required to decipher the detailed carcinogenic mechanism of UBE2S in such detected signaling pathways in cancers.

TME, composed of an extraordinary range of cellular subsets including tumor cells and immune cell, are thought to dramatically correlate with exacerbated cancer presentation and immunotherapeutic response. However, no convincing conclusion has been drawn on whether and how UBE2S promotes TME remodeling to favor tumor malignant phenotypes and immunotherapeutic resistance [29]. The result of our study demonstrated that UBE2S was negatively correlated with the immune score and stromal score, indicating that a higher UBE2S expression with a lower level of tumor-infiltrating immune and stromal cells may be an indicator of an advanced stage and unsatisfied prognosis. Hereafter, we further characterized the alteration of predominant cellular composition, finding that UBE2S had an extensive and complex regulatory relationship with surrounding tumor cells. Interestingly, although the negative regulatory trend was depicted in most cancers, we observed positive regulation of M1 macrophages and CD8+ T cells functioning as antitumor roles as well as negative regulation of CD4+ T cells and MDSCs (served as tumor-promoting roles including M2 macrophage, DC, and neutrophil) in some tumors. The correlation between the elevated UBE2S expression and the worse tumor phenotypes suggests that UBE2S may help tumor cells escape immune clearance through the interplay of immune cells with tumor cells. Hence, UBE2S may be responsible for TME reshaping and function as a double-edged sword across different tumors. Mechanistically, our work demonstrated a significant association of UBE25 with a series of immune regulatory molecules, especially immune checkpoint molecules, in most cancers analyzed herein. Our results highlight the upregulation of immunoinhibitors of immune checkpoint molecules including CD276, IDO1, LAG3, PDCD1, CD274, CTLA4, and TIGIT in LIHC, OV, TGCT, KIRP, and BLCA. Besides, the expression of cell exhaustion-related genes, including PDCD1, CTLA4, LAG3, and TIGIT, was upregulated in several tumors, indicating that UBE2S was intensely involved in tumor evasion via different mechanisms. Taken together, UBE2S may play a vital role in the TME and could confer tumor immune escape, although further investigation is needed to validate these conclusions.

Immunotherapy, especially immune checkpoint inhibitors, and targeted therapy have transformed cancer treatment and have emerged as well-established care for various human cancers in that they make complete and even durable responses possible even in patient with advanced cancers. However, only a narrow range of cancerous subpopulations could strikingly benefit from such treatments. Hence, it is essential to discover additional robust indicators for efficacy prediction and therapeutic target potential therapeutic targets. An increasing number of studies have addressed the importance of cancerous stemness and DNA methylation for drug sensitivity and resistance [22, 30, 31]. Beyond this, genome instability markers, including TMB, HRD, MSI, and MMR deficiency, were also reported to yield a profound impact on the immunotherapeutic response profiles [18–21]. Our current study discovered that upregulation of UBE2S dramatically promoted tumor stemness in nearly all analyzed cancers including LGG, TGCT, READ, LUAD, UCEC, DLBC, and BRCA. Similarly, the trend of UBE2S coexpression with TMB, HRD, MSI, and MMR was also depicted in our results, indicating that UBE2S might participate in cancerous developmental processes covering signaling, proliferation, and migration. Our analysis based on experimental evidence further corroborated that UBE2S overexpression conferred significant resistance to multiple targeted drugs in human tumors. The results obtained in this study suggest that UBE2S may serve as an integrated biomarker for efficacy and prognosis prediction instead of high-cost and complex traditional indicators.

To further confirm the reliability of the conclusion, we conducted in vitro validation to detect the association between UBE2S and HCC phenotypes. In accordance with aforementioned analyses, UBE2S knockdown attenuated the phenotypes of proliferation, clonogenicity, and migration in MHCC-97H cell, indicating the definite role of UBE2S in tumorigenesis and development.

Apart from the aforementioned key findings, there were several limitations in our study. Firstly, as the majority of analyses were performed based on the existing databases, further external experimental validation is warranted. In addition, as the data concerning RNA-sequencing, clinical stage and prognosis, enriched pathways, and drug sensitivity were retrieved from a variety of databases, analytic bias is inevitable.

In summary, few studies have described the immune-oncology pattern of UBE2S in human cancers. To the best of our knowledge, this is the first pan-cancer study reporting the association of aberrant UBE2S expression with the clinicopathological characteristics of patients with advanced-stage cancers and poor prognosis. In addition, we described the landscape of mutational features, DNA methylation, and RNA modification of UBE2S at gene and protein levels. Using enrichment analysis, we also summarized the potential pathophysiological functions and mechanisms of cancer development and progression and unveiled the substantial correlations of UBE2S with tumor infiltration of immune cells, immune checkpoint modules, and immunotherapeutic response-related genes, demonstrating that UBE2S played significant roles in immune evasion and could serve as a predictor of response to immunotherapy. All these findings may provide novel insights into the essential immune-oncology properties of UBE2S in the field of cancer research.

## 5. Conclusions

Taken together, UBE2S is associated with various cancerous phenotypes, including tumor proliferation, migration, prognosis, immune infiltration and evasion, and therapy response, which supports UBE2S as an immune-oncogenic molecule.

## Data Availability

Publicly available datasets were analyzed in this study. All relevant data can be found here: TCGA GDC Portal (https://portal.gdc.cancer.gov/), cBioPortal (http://www.cbioportal.org/), GEPIA2 (http://gepia2.cancer-pku.cn), TIMER2.0 (http://timer.cistrome.org/), and Cancer Cell Line Encyclopedia database (CCLE, https://sites.broadinstitute.org/ccle).
